# Role of Renal Dopamine Receptors in the Regulation of Blood Pressure

**DOI:** 10.3390/biom16040532

**Published:** 2026-04-02

**Authors:** Jian Yang, Pedro A. Jose

**Affiliations:** 1 Research Center for Metabolic and Cardiovascular Diseases, The Third Affiliated Hospital of Chongqing Medical University, Chongqing, 401120, China; 2 Department of Clinical Nutrition, The Third Affiliated Hospital of Chongqing Medical University, Chongqing, 401120, China; 3 Division of Renal Diseases & Hypertension, Departments of Medicine and Pharmacology/Physiology, The George Washington University School of Medicine & Health Sciences, Washington, DC 20052, USA

**Keywords:** dopamine receptor, blood pressure, hypertension, kidney

## Abstract

Hypertension continues to be a major global public health challenge. Dopamine generated in the kidney is a vital coordinator of sodium homeostasis and blood pressure control. Dopamine exerts its effects by activating its receptors, which are divided into the D_1_-like receptor family (D_1_R and D_5_R) and the D_2_-like receptor family (D_2_R, D_3_R, and D_4_R). All five dopamine receptor subtypes are differentially expressed along the nephron. Dopamine receptors inhibit the activities and/or expression of renal tubular sodium transporters/exchangers/channels, decrease renal oxidative stress, and interact with other receptors, including angiotensin II receptors. Many studies have demonstrated that renal dopamine receptors play an important role in the regulation of blood pressure. The germline deletion or renal-selective silencing of any of the five dopamine receptor subtypes may impair sodium excretion and increase blood pressure. In addition, renal dopamine receptor expression and/or function are regulated by some factors such as G protein-coupled receptor kinases, oxidative stress, and sorting nexins. In this article, we summarize the role of each dopamine receptor subtype in the pathogenesis of hypertension and discuss the potential regulatory mechanisms of their expression and function. These may lead to the development of novel therapeutic approaches to the prevention and treatment of hypertension.

## 1. Introduction

Essential hypertension, also known as primary hypertension, is the persistent abnormal increase in systolic blood pressure and/or diastolic blood pressure. It is recognized as one of the leading causes for all-cause morbidity and a significant risk factor for cardiovascular and other diseases, such as stroke and end-stage renal disease [1]. According to the first global report on hypertension of the World Health Organization in 2023, the number of people with hypertension doubled from 650 million in 1990 to 1.3 billion in 2019 [2]. The prevalence of hypertension in low- and middle-income countries increased in both urban and rural areas between 1990 and 2020 [3]. Even in developed countries, such as the US, the prevalence of hypertension among adults is estimated to be 46.7%, which equates to about 122.4 million subjects [4]. Thus, how to prevent and treat hypertension has become a major global public health challenge.

The pathogenesis of hypertension is complex, determined by genetic, lifestyle, and environmental factors that interact to increase blood pressure and cause end-organ damage [5]. It is well-known that aberrant sodium metabolism, causing excessive sodium in the body, is an important risk factor for the occurrence of hypertension. The kidney is a vital organ in the long-term regulation of blood pressure through maintenance of normal sodium homeostasis [6]. The increased sodium retention in hypertension is, at least in part, due to increased activity of renal sodium transporters/exchangers/pump/channels *per se* and/or the abnormal regulation of sodium transport [7,8]. The maintenance of a normal balance of natriuresis and anti-natriuresis is regulated by endocrine factors. Among these, dopamine, produced in the kidney, as a vital coordinator of sodium homeostasis and blood pressure through an independent intrarenal dopaminergic system, has attracted much attention [9].

Dopamine, also known as 3-hydroxytyramine, plays an essential role as an endogenous neurotransmitter catecholamine in the central nervous system. Moreover, dopamine also exerts physiological functions in other organs, including the artery, stomach/intestine, kidney, liver, and systems, such as the immune system [10,11,12,13]. Among them, the kidney has received considerable attention due to its ability to regulate dopamine production and its local renal effects. Dopamine exerts its effects by activating its five receptors. Many studies have demonstrated that renal dopamine receptors play an important role in the regulation of blood pressure [14,15,16,17]. In this review, we discuss the classification of dopamine receptors and their biological functions in the kidney, summarize the role of each dopamine receptor subtype in the pathogenesis of hypertension, and then discuss the potential regulatory mechanisms on their expression and function. This may promote our understanding of the role of renal dopamine receptors in the regulation of blood pressure and propose novel therapeutic approaches for the prevention and treatment of hypertension.

## 2. Intrarenal Dopaminergic System

The kidney synthesizes dopamine, independent of renal nerves, and has an intrarenal dopaminergic system. The circulating dopamine concentration is usually in the picomolar range, which is not high enough to activate its receptors. However, dopamine levels in specific segments of the kidney, such as renal proximal tubules (RPTs), can reach high nanomolar concentrations [17]. Unlike neural cells, RPT cells cannot produce the dopamine precursor L-dihydroxyphenylalanine (L-DOPA), and the dopamine produced in the kidney is not converted to norepinephrine because dopamine β-hydroxylase is not expressed in RPT cells [18]. Filtered L-DOPA is taken up by RPT cells via sodium-dependent and sodium-independent amino acid transporters and then metabolized to dopamine by aromatic amino acid decarboxylase (AADC) [19]. The selective deletion of AADC in RPTs in mice reduces intrarenal dopamine levels, increases the expression of renal sodium transporters/exchangers and water channel, including sodium hydrogen exchanger type 3 (NHE3), sodium-bicarbonate cotransporter (NBC), solute carrier family 12, member 1 (NKCC2), solute carrier family 12, member 3 (NCC), and aquaporin 2 (AQP2), and causes salt-sensitive hypertension [20].

The renal tubular synthesis/release of dopamine is regulated by several factors, such as dietary salt and intracellular sodium [21,22]. When dietary salt intake is increased, the intrarenal dopaminergic system is estimated to be responsible for over 50% of the increase in urinary sodium and water excretion. The increase in renal sodium excretion due to dopamine may be, at least in part, caused by inhibition of sodium transporter, exchanger, and pump activities, in the short-term, and a decrease in the expression of several sodium transporters, exchangers, and pumps, in the long-term. However, the generation of dopamine by the kidney and the dopaminergic-mediated natriuretic response to an acute sodium load are impaired in some hypertensive subjects [23]. In addition, animal studies have shown that the inhibition of renal dopamine production by carbidopa, an inhibitor of peripheral dopa decarboxylase, increases the blood pressure of spontaneously hypertensive rats (SHRs), which is accompanied by a decrease in urinary sodium and dopamine excretion [24]. Under physiological states, enhanced sodium intake *per se* increases the renal generation of dopamine, which may be responsible for the increased urinary sodium excretion or a compensatory response for defective renal dopamine receptors. However, the combination of increased sodium intake and oxidative milieu accelerates renal dopamine oxidation and inflammation, subsequently causing hypertension [25]. These suggest that a dysfunction of the intrarenal dopaminergic system is involved in the pathogenesis of hypertension.

## 3. Renal Dopamine Receptor Subtypes

In mammals, intrarenal dopamine exerts its physiological effects via a class of cell surface receptors, which belong to the α-group of the rhodopsin-like (Class A) family of seven transmembrane receptor proteins, called G protein-coupled receptors (GPCRs) [26]. Based on their molecular structure and pharmacology, dopamine receptors are divided into two subfamilies: the D_1_-like and the D_2_-like receptor families. The D_1_-like receptors, comprised of dopamine D_1_ receptor (D_1_R) and dopamine D_5_ receptor (D_5_R) subtypes, couple to stimulatory G-proteins (Gαs and G_olf_) and stimulate the activity of adenylate cyclase to increase cytosolic cAMP production [27]. The D_2_-like receptors, composed of dopamine D_2_ receptor (D_2_R), dopamine D_3_ receptor (D_3_R), and dopamine D_4_ receptor (D_4_R) subtypes, couple to inhibitory G proteins (Gαi and Go), and inhibit adenylyl cyclase activity [18].

All five dopamine receptor subtypes are expressed in the mammalian nephron. However, the dopamine receptor subtypes have distinct distributions in the nephron [15]. All five subtypes of the dopamine receptor family are expressed in the RPT. Three dopamine receptor subtypes, D_1_R, D_3_R, and D_5_R, are expressed in the thick ascending limb of Henle. All the dopamine receptor subtypes, except the D_2_R, are expressed in the distal convoluted tubule. However, the collecting duct expresses all subtypes of the dopamine receptors, except D_3_R. Currently, studies have shown that all five dopamine receptor subtypes are involved in the regulation of urinary sodium excretion and blood pressure [9,15,18,28,29]. Aberrant expression and/or dysfunction of renal dopamine receptors disturb sodium homeostasis and cause the development of hypertension (Table 1).

## 4. Renal Dopamine Receptors and Blood Pressure Regulation

### 4.1. Renal D_1_R-Mediated Blood Pressure Regulation

#### 4.1.1. Physiological Effects of Renal D_1_R

The stimulation of D_1_-like dopamine receptors decreases tubular sodium reabsorption and promotes urinary sodium excretion. The intrarenal infusion of fenoldopam, an agonist of D_1_-like receptors, induces natriuresis and diuresis in normotensive Sprague–Dawley (SD) rats fed normal or high salt diets [30,31]. During normal sodium intake, the D_1_-like dopamine receptors are responsible for 50–70% urinary sodium excretion. It is difficult to determine whether the D_1_R and/or the D_5_R mediate this effect because there are no available specific ligands that can distinguish the two receptor subtypes (see below). However, it is generally believed that the natriuretic effect of D_1_-like dopamine receptors in the proximal tubule is primarily attributed to the D_1_R since the D_1_R accounts for almost all of the cAMP production after D_1_-like receptor activation [18]. D_1_R-specific gene silencing inhibits cAMP accumulation induced by activation of D_1_-like receptor, whereas either a novel D_5_R-selective antagonist LE-PM436 or D_5_R siRNA has no effect on D_1_-like receptor-mediated increase in cAMP production [32]. Moreover, D_1_R depletion, using D_1_R siRNA, blocks the D_1_-like receptor-dependent inhibition of sodium transport [32]. This is confirmed by renal D_1_R knockdown. Intrarenal administration of D_1_R antisense oligonucleotides (AS-ODN) reduces urinary sodium excretion and urine output, but does not affect systolic blood pressure, in female SD rats on a normal or high salt diet [33]. Moreover, global ablation of the *Drd1* gene in mice also increases systolic blood pressure [34]. The possible reasons for the heterogeneity of phenotypes of blood pressure may be due to (1) AS-ODN-induced antinatriuresis is short-lasting, which is for only two days on a normal salt diet and three days on a high salt diet. Longer term suppression of the renal D_1_R may be necessary to show its effect on the regulation of blood pressure; (2) AS-ODN only reduces renal D_1_R protein by 46% [33]. Complete knockout or more complete suppression of D_1_R may be needed to change blood pressure; (3) blood pressure was measured by the noninvasive tail-cuff method in AS-ODN studies, but arterial blood pressure was recorded from the femoral artery in D_1_R knockout mice.

D_1_-like receptor-mediated natriuretic effect is, at least in part, due to direct inhibition of the activities of renal tubular sodium transporters. The activation of D_1_-like receptors inhibits the activities of basolateral sodium bicarbonate cotransporter and Na^+^-K^+^-ATPase (NKA), and luminal NHE3, sodium phosphate cotransporter type 2 (NaPi2), chloride bicarbonate (Cl^-^/HCO3^-^) exchanger, and the epithelial sodium channel (ENaC) [32,35]. These pharmacological studies were confirmed using D_1_R siRNA, which prevented the inhibition of sodium transport induced by D_1_-like receptor activation [35]. The D_1_R-mediated natriuretic effect is also related to its negative regulation of oxidative stress. Stimulation of D_1_R reduces renal nicotinamide adenine dinucleotide phosphate (NADPH) oxidase activity and subsequently inhibits renal oxidative stress [36], which is involved in the positive regulation of sodium transporters and blood pressure [70]. In addition, the renal D_1_R also interacts with other systems such as the renin-angiotensin-aldosterone system (RAAS) and other renal receptors, including the uroguanylin receptor, angiotensin II (Ang II) receptors, to regulate, cooperatively, urinary sodium excretion and maintenance of sodium homeostasis [37,38,71].

#### 4.1.2. Renal D_1_R in Hypertension

The human *DRD1* gene locus on chromosome 5 at q35.1 is linked to human essential hypertension [72]. *DRD1* polymorphism A-48G is associated with essential hypertension in Japanese. Single-nucleotide polymorphisms (SNPs) (rs1799914 and rs4867798) of the *DRD1* gene are also associated with hypertension in Hani Chinese [73]. A meta-analysis showed that the rs4532 locus of the *DRD1* gene is associated with hypertension in East Asians [74].

Impaired D_1_R-mediated renal effect is involved in the development of hypertension. The natriuretic and diuretic responses to D_1_-like receptor agonists are impaired in different animal models of hypertension [39,40,41]. Moreover, the ability of D_1_-like receptors to inhibit renal sodium transport is also impaired in hypertensive humans [42]. The impaired D_1_-like receptor function in hypertension may be due, in part, to increased D_1_R serine phosphorylation and subsequent uncoupling of the D_1_R from its G protein/effector complex in RPTs [43,44], which leads to the attenuated inhibition of the activity of sodium transporters [75]. Renal D_1_R expression is also decreased in some animal models of hypertension [45,46,47]. The reduced D_1_R expression and/or function leads to increased oxidative stress and aberrant interaction between D_1_R and other receptors, which decreases urinary sodium excretion and subsequently the development of hypertension [37,38,71].

### 4.2. Renal D_2_R-Mediated Blood Pressure Regulation

#### 4.2.1. Physiological Effects of Renal D_2_R

The D_2_R exerts many physiological functions in the kidneys, including inhibiting sodium transport, suppressing renal inflammation, preventing ischemia/reperfusion injury, and decreasing renal fibrosis [51,76,77,78]. Moreover, D_2_R synergistically interacts with the renal D_1_R to increase sodium excretion in SD rats [49]. Although *Drd2* knockout mice have increased systolic and diastolic blood pressures, these may not be due to impairment of renal sodium excretion [79]. However, another study found that the global ablation of the *Drd2* gene in male mice causes a salt-dependent increase in blood pressure [80]. In addition, in mice, the renal cortical *Drd2* depletion with siRNA reduces renal sodium excretion and increases blood pressure, which are reversed by renal-selective D_2_R rescue [81].

The D_2_R-mediated sodium excretion is associated with its negative regulation of renal oxidative stress. The D_2_R decreases renal reactive oxygen species (ROS) production by reducing NADPH oxidase expression and activity and increasing the expression of antioxidant enzymes such as sestrin2 [50]. These indicate that D_2_R-mediated regulation of oxidative stress involves both pro-oxidant and antioxidant systems. The D_2_R also regulates renal sodium transport by inhibiting NKA and NHE3. The D_2_R directly inhibits adenylyl cyclase-mediated cAMP production, which activates NKA [82]. However, the simultaneous stimulation of D_1_R and D_2_R is needed for dopamine to inhibit NKA activity in the RPT [48]. Similarly, D_1_R and D_2_R synergistically increase NHE-3 phosphorylation, where D_2_R, by itself, is ineffective [83]. In addition, D_2_R increases urinary sodium excretion by increasing the synthesis of dopamine in the kidney [51].

It should be noted that D_2_R has effects in both renal tubules and presynaptic sympathetic ganglia. However, the norepinephrine excretion in *Drd2* knockout mice was reduced during the high-salt diet period [80]. This may suggest that the inhibition of sympathetic tone was accompanied by sodium retention and increased blood pressure, which may exclude the possibility that the sympathetic activation is responsible for the high-salt induced changes in sodium balance and blood pressure. In addition, heart rate, another indicator of sympathetic activity, was not different between *Drd2* knockout and wild-type (WT) mice. These indicate that the sodium retention and increased blood pressure in *Drd2* knockout mice are primarily due to renal tubular dysregulation. However, increased sympathetic activity may also be involved in the D_2_R-mediated regulation of blood pressure. α-adrenergic blockade decreased blood pressure to a greater extent in *Drd2* knockout than WT mice, and epinephrine excretion was greater in *Drd2* knockout than WT mice [79]. Moreover, the interaction of the sympathetic nervous system and RAAS is also important in increasing blood pressure levels in *Drd2* knockout mice.

#### 4.2.2. Renal D_2_R in Hypertension

The *DRD2* gene is located on chromosome 11, which is linked to human essential hypertension [84]. A TaqI polymorphism of the *DRD2* gene is associated with increased blood pressure [85]. Another study showed that a polymorphism in exon 6 of the *DRD2* gene is associated with hypertension [86]. The plasma membrane D_2_R expression is decreased in urine-derived RPT cells isolated from subjects with inverse salt sensitivity, a condition in which a low sodium intake increases blood pressure, relative to those cells isolated from salt-resistant subjects (subjects whose blood pressures are not affected by changes in salt intake), that may be caused by *DRD2* variants (rs6276 and 6277) [52]. However, the renal cellular immunolocalization and protein expression of D_2_R are not different between SHR and WKY rats [61]. Nevertheless, the renal D_1_-like and D_2_-like receptor interaction is impaired in SHRs, which causes reduced natriuresis and diuresis [53].

### 4.3. Renal D_3_R-Mediated Blood Pressure Regulation

#### 4.3.1. Physiological Effects of Renal D_3_R

Similar to D_1_-like and D_2_ receptors, the activation of renal D_3_R increases sodium excretion. The intrarenal arterial infusion of PD128907, a D_3_R agonist, induces natriuresis and diuresis in normotensive rats [58]. In Dahl salt-resistant rats fed either a normal or high-sodium diet, another D_3_R agonist, 7-hydroxydipropyl-aminotetralin (7-OH-DPAT), also increases urinary sodium excretion. However, although 7-OH-DPAT induces natriuresis in salt-sensitive Dahl rats fed with a normal diet, this does not occur in those fed a high-salt diet [59]. The impaired ability to excrete a sodium load is also present in D_3_R knockout mice. Disruption of the *Drd3* gene in mice increases renal renin levels, and renal Ang II type 1 receptor (AT_1_R) expression, induces sodium retention, and subsequently causes renin-dependent hypertension [54,56,87]. However, another study reported that male *Drd3* knockout mice do not display the hypertensive phenotype regardless of salt intake, although urinary sodium excretion is decreased in these male *Drd3* knockout mice fed a high salt diet [88].

The reason that the *Drd3* knockout mice show different blood pressure phenotypes may not be due to genetic background, because the *Drd3* knockout mice in all four studies are on a C57B1/6J background. Therefore, the possible reasons for the discrepancy among the different studies may be due: (1) sodium protocol: one study used mice on a normal salt diet (0.8% NaCl) [56], one study used a long-term cross-over experiment (low salt diet, 0.2% NaCl; high salt diet, plus 1.0 mg/kg body wt. deoxycorticosterone acetate) [88], and sodium intake was not stated in the other two studies [54,87]; (2) different methods of blood measurement: blood pressure was measured via the femoral vessels in three studies [54,56,87], but tail-cuff plethysmography was used in another study [88]; (3) animal age and sex: the animal age was 3 months in two studies [87,88], but not stated in the other two studies [54,56]; only one study reported that the experiments were performed in male mice [88], but the sex of the animas was not stated in the other studies [54,56,87]; (4) state of consciousness: three studies reported that the mice were anesthetized with pentobarbital (50 mg/kg intravenously) [54,56,87], but another one was performed in conscious mice [88].

D_3_R-mediated renal sodium excretion is partly attributed to its ability to inhibit renal tubule sodium transport, related to NKA, NHE, and NHE3 [57]. The D_3_R also regulates the expression of NHE3. Stimulation of D_3_R inhibits the deubiquitinylating activity of ubiquitin-specific peptidase 48, which promotes NHE3 degradation and subsequently reduces sodium transport; pharmacological blockade of D_3_R increases renal NHE3 expression [56]. Moreover, the renal D_3_R is involved in the regulation of renal hemodynamics [60]. In deoxycorticosterone acetate (DOCA)-salt hypertensive rats, the intrarenal medullary blockade of D_3_R increases mean arterial pressure in parallel with impairment of renal hemodynamics [89]. In addition, renal D_3_R interacts with other receptors such as other dopamine receptors (i.e., D_5_R), endothelin B receptor, and Ang II receptors, to maintain a normal sodium homeostasis [54,55,58].

#### 4.3.2. Renal D_3_R in Hypertension

The chromosome locus of the *DRD3* gene (3q13.3) is linked to human essential hypertension [90]. Soma et al. reported that there is no association between the Ser9Gly polymorphism in the D_3_R gene or other D_3_R gene variants with hypertension [91]. However, another study showed that SNPs (rs9880168) of the *DRD3* are associated with essential hypertension in subjects of Hani nationality, but not in subjects of Han and Yi nationality, in China [92].

Compared with WKY rats, SHRs fed a normal-salt or high-salt diet have reduced D_3_R expression in the renal cortex and RPT cells [54,61]. There are no differences in D_3_R expression in the inner medulla of WKY rats and SHRs [61]. However, the D_3_R agonist PD128907-induced increase in D_3_R expression is not evident in RPT cells from SHRs [54]. These are reflected by a decrease in D_3_R physiological actions. The renal D_3_R-mediated sodium excretion is impaired in SHRs on a normal-salt or on a high-salt diet, relative to WKY rats [58]. In addition, the impaired natriuretic effect induced by activation of D_3_R in SHRs is also associated with the aberrant interaction of D_3_R and other receptors [54,55,58].

### 4.4. Renal D_4_R-Mediated Blood Pressure Regulation

#### 4.4.1. Physiological Effects of Renal D_4_R

The stimulation of renal D_4_R increases urinary sodium excretion in WKY rats [62]. Moreover, renal D_4_R has been shown to inhibit vasopressin-dependent water and sodium reabsorption in the cortical collecting duct [63,93]. In the rabbit cortical collecting duct, it is the basolateral, not luminal D_4_R, that is mainly responsible for its natriuretic action [94]. The absence of the *Drd4* gene in both male and female mice increases blood pressure by increasing renal AT_1_R expression [95]. There are no differences in serum aldosterone concentration, plasma renin concentration, and urinary dopamine between *Drd4* knockout mice and their littermates [95,96]. *Drd4* knockout mice on a normal or high sodium diet have increased blood pressure. The pressure-natriuresis curve is shifted to the right in male *Drd4* knockout mice, relative to WT mice. However, low sodium diet decreases blood pressure in both mouse strains [96].

D_4_R-mediated sodium excretion is partly due to the inhibition of the expression and/or activities of renal tubular sodium transporters. The D_4_R agonist PD168077 inhibits NKA activity in RPT cells from WKY rats [62]. *Drd4* knockout mice on a normal salt diet have increased renal expression of NHE3, NKCC2, NCC, and outer medullary α-ENaC. Moreover, relative to WT littermates, *Drd4* knockout mice on a high salt diet have increased expression of sodium transporters in the plasma membrane, which may mediate the salt sensitivity of *Drd4* knockout mice [96]. These alterations of the expression of sodium transporters may be due to the ability of D_4_R to regulate the phosphorylation or degradation of sodium transporters [96,97]. Moreover, renal D_4_R also interacts with other renal receptors, including AT_1_R, insulin receptor, and other dopamine receptors, to regulate urinary sodium excretion [62,64,65].

#### 4.4.2. Renal D_4_R in Hypertension

A locus of the *DRD4* gene (11p15.5) is linked to hypertension. The *DRD4* gene has a 16 amino acid (48 bp) repeat polymorphism located in exon 3, where a G-protein binding area is encoded. Studies have shown a correlation between an SNP and blood pressure. In a white population with 479 female and 385 male subjects, the long variant of the *DRD4* gene is associated with a 3 mm Hg higher systolic and 2 mm Hg higher diastolic blood pressure, relative to non-carriers of the long *DRD4* variant [98].

The D_4_R protein expression is increased in the renal cortex and RPT cells of SHRs, relative to WKY rats. However, there is no difference in the D_4_R protein expression in the inner medulla between WKY rats and SHRs [61,64]. The phosphorylation of D_4_R is higher in SHR RPT cells than in WKY cells [62]. Moreover, the D_4_R-induced diuretic and natriuretic effects are impaired in SHRs, relative to WKY rats [64]. In addition, the aberrant interaction between D_4_R and other renal receptors is also involved in the impaired urinary sodium excretion and increased blood pressure [62,64,65].

### 4.5. Renal D_5_R-Mediated Blood Pressure Regulation

#### 4.5.1. Physiological Effects of Renal D_5_R

The D_5_R, which also belongs to the D_1_-like receptor subgroup, has an 80% homology in its transmembrane domain and a 30% homology in its N and C termini with the D_1_R [99]. However, D_5_R has characteristics that are distinct from D_1_R. For example, D_5_R has a 10-fold higher affinity for dopamine than D_1_R; D_5_R has constitutive activity, which increases the basal activity of adenylyl cyclase [100,101]. These indicate the potential role of renal D_5_R in the basal regulation of sodium homeostasis and blood pressure.

The role of the renal D_5_R in the regulation of blood pressure has been verified in *Drd5* knockout mice, which have increased systolic and diastolic blood pressures on a normal salt diet [102]. This is further confirmed by cross-transplantation studies. A kidney lacking D_5_R transplanted into a WT mouse increased systolic and diastolic blood pressures, while a kidney from a WT mouse transplanted into a mouse lacking the D_5_R decreased blood pressure [66]. These suggest the important role of renal D_5_R in the regulation of blood pressure. Moreover, the increased blood pressure in *Drd5* knockout mice is further increased on a high salt diet, indicating the salt sensitivity of blood pressure in *Drd5* knockout mice [66,68,102].

Similar to the other dopamine receptor subtypes, the regulation of blood pressure by the renal D_5_R is associated with its regulation of renal sodium transport. Activation of D_5_R by fenoldopam reduces NKA activity in WKY RPT cells with D_1_R depletion or in D_5_R-transfected HEK293 cells [55]. Moreover, D_5_R and D_1_R, via a D_1_R/D_5_R heteromer, synergistically inhibit NHE3 and NKA activities and reduce renal sodium transport [32]. *Drd5* knockout mice have higher renal protein expressions of NKCC2, NCC, and ENaC on a normal salt diet, which persist when they are fed a high salt diet [102]. Moreover, the renal protein levels of NHE3 and NaPi2 are also increased in *Drd5* knockout mice that are fed a high salt diet [102]. It should be noted that although both D_1_R and D_5_R have similar functions, D_5_R has unique functions distinct from D_1_R. For example, the increased expression of some renal sodium transporters on a normal salt diet persists, while the high blood pressure is increased further when *Drd5* knockout mice are fed a high salt diet [68,102], which is not observed in *Drd1* knockout mice. The effects of all five dopamine receptor subtypes modification on renal function and blood pressure are presented in Table 2.

**Figure 1:**
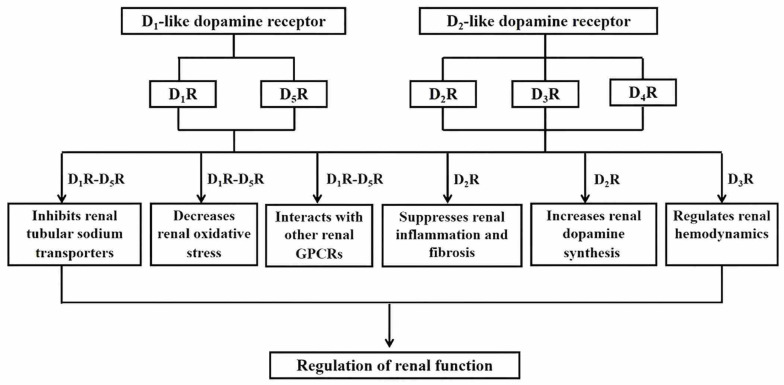
The proposed mechanisms by which dopamine receptor subtypes regulate renal function.

D_5_R-mediated anti-oxidant stress effect is another important mechanism by which renal D_5_R regulates blood pressure. D_5_R directly decreases NADPH oxidase protein expression and activity and inhibits ROS production [68]. These were confirmed in *Drd5* knockout mice, which have increased NADPH oxidase protein expression and activity and ROS production [68]. The increased blood pressure and oxidant stress in *Drd5* knockout mice are normalized by treatment with apocynin, an NADPH oxidase inhibitor [68]. Cross-transplantation studies between *Drd5* knockout and WT mice showed that a kidney from a WT mouse transplanted into a mouse lacking D_5_R decreases renal NADPH oxidase isoform 2 (NOX2) expression, while a kidney lacking D_5_R transplanted into a WT mouse increases renal NOX2 expression [66]. D_5_R exerts its natriuretic effect by other mechanisms. For example, D_5_R antagonistically interacts with other receptors (e.g., AT_1_R) to maintain normal blood pressure [69]. The proposed mechanisms by which dopamine receptor subtypes regulate renal function are presented in Figure 1.

#### 4.5.2. Renal D_5_R in Hypertension

The locus of DRD5, 4p15.1-16.1, is linked to human essential hypertension. D_5_R SNPs are involved in the regulation of blood pressure. Humans have SNPs in the *DRD5* gene, some of which decrease D_5_R function [103]. The human D_5_RF173L (hD_5_RF173L) mutation reduces D_5_R-mediated cAMP generation. This is reflected in its physiological function; male hD_5_RF173L transgenic mice have increased blood pressure, which is accompanied by impaired natriuresis and increased renal AT_1_R expression [67]. These are related to reduced renal expression of Trx1, an antioxidant, and increased renal NADPH oxidase activity and ROS generation [67].

Renal D_5_R expression is decreased in different hypertensive animal models. Basal D_5_R levels are reduced in SHR RPT cells and renal brush border membranes of SHRs [69]. There is interaction between D_5_R and other receptors expressed in the kidney, impairment of which can lead to decreased natriuresis and hypertension [69].

## 5. Regulation of Renal Dopamine Receptor Expression and Function

### 5.1. GRK Regulation of Dopamine Receptors

The GPCR kinase (GRK) family has seven members, which are characterized by their ability to recognize and phosphorylate agonist-occupied GPCRs, including dopamine receptors [104,105]. Among the seven GRK subtypes, GRK4 has attracted increased attention because of its important role in the regulation of blood pressure. GRK4 is expressed in only a few tissues, i.e., brain, kidney, myometrium, and testis. GRK4 is well-expressed in the subapical membranes of RPTs and thick ascending limbs of Henle and arteries in both WKY and SHRs [106,107]. GRK4 constitutively phosphorylates GPCRs in the absence of agonist activation [108].

Most current studies on GRK4 have focused on its regulation of D_1_R. Compared with WKY rats, SHRs have higher basal serine-phosphorylated D_1_R in RPTs, brush border membranes, and membranes from RPT cells, which are accompanied by greater renal GRK4 expression [16]. The depletion of renal GRK4 with AS-ODN in SHRs increases sodium excretion and urine volume, causes a marked decrease in the increased arterial blood pressure, as well as a decrease in the increased serine-phosphorylated levels of renal D_1_R [16]. GRK4 silencing completely prevents the serine phosphorylation of D_1_R and restores fenoldopam-mediated cAMP accumulation in RPT cells from hypertensive subjects [109]. GRK4 expression is increased in obese Zucker rats, which causes hyperphosphorylation of D_1_R and their uncoupling from Gs proteins [110].

Several GRK4 SNPs, such as R65L, A142V, and A486V, have attracted attention for their important role in the pathogenesis of hypertension. Studies in vitro have shown that GRK4 SNPs increase the basal phosphorylation of D_1_R and markedly impair the D_1_R-induced cAMP generation [109]. This is reflected in in vivo studies. Transgenic mice expressing GRK4 A142V are hypertensive, in which the infusion of fenoldopam fails to increase urine flow and sodium excretion [109].

GRK2 is another GRK subtype that is involved in the regulation of renal dopamine receptors [111]. GRK2 expression and/or membranous translocation are increased in RPTs, which increase D_1_R phosphorylation and cause their uncoupling from signaling proteins in different hypertensive animal models [110,112]. GRK2 siRNA or AS-ODN abrogates D_1_R serine phosphorylation and normalizes D_1_R expression and affinity in insulin-treated opossum kidney cells and blunts the desensitization of the D_1_R in human RPT cells [113].

### 5.2. Dopamine Receptors Are Regulated by Oxidative Stress

Oxidative stress, one of the fundamental contributors to the pathogenesis of hypertension, occurs when ROS exceeds the capacity of antioxidant defense systems [114]. Excessive levels of ROS disturb the equilibrium between ROS and antioxidants, leading to the dysfunction of some organs and promoting the development of hypertension [115]. Among the organs regulating blood pressure, the kidney has gained attention as an important organ affected by oxidative stress [116,117].

In rat RPT cells, hydrogen peroxide, an oxidant, increases the levels of malondialdehyde, a marker of oxidative damage, which is accompanied by serine- enhanced phosphorylation of D_1_R, reduced membranous D_1_R expression, and its inhibition of NKA activity [118]. This is reflected in in vivo studies. L-buthionine sulfoximine increases oxidative stress, which increases D_1_R phosphorylation and defective D_1_R-G protein coupling, impairing D_1_R-mediated natriuretic response that subsequently leads to hypertension. Treatment with tempol, a superoxide scavenger, decreases oxidative stress, restores D_1_R-regulated renal function, and normalizes blood pressure [47]. The inhibition of nuclear factor erythroid 2-related factor 2 aggravates oxidative stress and inflammation, impairs renal D_1_R-induced natriuresis, which contributes to the development of hypertension in mice [119]. These indicate that normalization of renal D_1_R-mediated natriuretic function by reduction of oxidative stress may be a potential target in the treatment of hypertension.

Renal oxidative stress decreases renal D_1_R expression and/or increases D_1_R phosphorylation levels, impairs D_1_R-mediated natriuresis and diuresis, and increases blood pressure in several hypertensive animal models, including age-associated hypertension, hyperinsulinemia-mediated hypertension, and hypertension in offspring induced by maternal exposures to adverse agents or conditions, such as lipopolysaccharides, fine particulate matters, and maternal diabetes mellitus [112,120]. The detrimental role of oxidative stress in the regulation of D_1_R expression and its-mediated function has been verified by administration of antioxidants. The inhibition of ROS production by tempol decreases oxidative stress and normalizes blood pressure, accompanied by an increase in the low renal D_1_R expression and a decrease in hyperphosphorylated D_1_R [112,120].

Lifestyle is implicated in the development of hypertension [121]. Exercise, as a fundamental factor of lifestyle intervention, exerts many beneficial physiological effects in the regulation of blood pressure [122]. In animals with age-related hypertension, treadmill exercise decreases oxidative stress, restores renal D_1_R levels in RPT membranes, increases D_1_R-G protein coupling, and leads to increased sodium excretion [123]. However, another study found that although exercise reduces renal oxidative stress, it does not alleviate impaired renal D_1_R dysfunction and decrease the high blood pressure in obese Zucker rats, another hypertensive animal model [124]. These studies suggest that the role of oxidative stress in the regulation of D_1_R function may be different among different hypertensive animal models.

### 5.3. Regulation of Dopamine Receptor Trafficking

Sorting nexins (SNXs) are a diverse group of proteins orchestrating the trafficking of cargo in the cytoplasm and plasma membrane [125,126]. Our previous studies have shown that deficiency of SNXs disturbs the sorting and trafficking of dopamine receptors, impairs receptor-mediated functions, and increases blood pressure [127].

SNXs play a vital role in the trafficking and signaling of renal D_1_R. SNX5 colocalizes and dynamically interacts with D_1_R in the human kidney and RPT cells. SNX5 depletion inhibits agonist-induced D_1_R endocytosis and cAMP production, and delays D_1_R recycling. SNX5 also interacts with GRK4, which restrains GRK4 from targeting the phosphorylation of D_1_R [127]. Renal SNX5 silencing in SHRs results in a further increase in blood pressure, which is accompanied by a decrease in sodium excretion [127]. Another SNX, SNX19, is also involved in the regulation of renal D_1_R. SNX19, which contains caveolin-1 and flotillin-1 binding motifs, plays an important role in the localization and trafficking of the D_1_R to lipid raft microdomains [128]. Renal Snx19 silencing decreased renal D_1_R expression and increased the systolic blood pressure of C57BL/6J mice [128].

The D_5_R is also regulated by another SNX subtype, i.e., SNX1. Renal SNX1 interacts with D_5_R and is required for its trafficking. SNX1 depletion in human RPT cells abrogates renal D_5_R-induced cAMP production and inhibition of sodium transporter activity [129]. The SNX1 and D_5_R interaction in vitro is reflected in animal studies. Renal-selective SNX1 silencing in C57BL/6J and BALB/cJ mice increases blood pressure and blunts D_5_R-mediated natriuretic response [129]. Renal SNX1 depletion also increases renal AT_1_R expression, which is involved in the dysfunction of D_5_R [69,129]. *Snx1* knockout mice have increased blood pressure, accompanied by increased renal ROS production and impaired D_5_R function, which can be reversed by treatment with antioxidants [130]. In addition, some SNPs of the SNX1 gene are associated with a decrease in systolic blood pressure in response to the diuretic hydrochlorothiazide in hypertensive African-Americans [130]. These studies suggest that the disturbed regulation of SNXs in the trafficking of renal dopamine receptors leads to impaired receptor-mediated sodium excretion and causes hypertension. Trafficking proteins, such as SNX, may be a potential target for the treatment of hypertension.

## 6. Conclusions and Perspectives

In summary, increasing evidence shows that dopamine receptors play important roles in the regulation of sodium balance and blood pressure. All five dopamine receptor subtypes are expressed in the kidney with distinct distributions in the nephron. Dopamine receptors inhibit the activities and/or expression of renal tubular sodium channels, exchangers, transporters, and pumps, decrease oxidative stress, and interact with other GPCRs. Among the above mechanisms, dopamine receptor-mediated inhibition of renal tubular sodium channels may be the primary mechanism, while the dopamine receptor-mediated regulation of oxidative stress and interaction with other receptors may be secondary. It should be noted that oxidative stress also impairs the expression and function of dopamine receptors. Thus, antioxidants could restore the impaired receptor expression and function in hypertension.

Knockout of any of the five dopamine receptor subtypes may decrease sodium excretion and increase blood pressure. These reports demonstrate that aberrant expression and/or dysfunction of any of the dopamine receptors in the kidney may play an important role in the pathogenesis of hypertension. Thus, further studies targeting specific dopamine receptor subtypes or related regulatory factors may provide new therapeutic antihypertensive strategies in the future.

However, it should be noted that current studies that demonstrate the possible role of renal dopamine receptors in the regulation of blood pressure are mostly based on animal studies and in vitro experiments. There are few human studies in this field, which have only focused on the relationship between SNPs of dopamine receptors and hypertension. Results from animal studies cannot establish a causal relationship in humans. Therefore, more clinical evidence is needed in the future to support the role of renal dopamine receptors in the regulation of blood pressure.

## Figures and Tables

**Table 1:** Summary of renal dopamine receptors-mediated physiological effects and their abnormities in hypertension.

Receptor Subtype	Distribution in the Kidney	Physiological Effects in the Kidney	Aberrant Expression and/or Function in Hypertension
D_1_R (*DRD1*)	Renal proximal tubule, thick ascending limb of Henle, distal convoluted tubule, cortical collecting duct	Inhibits renal sodium transport and increases sodium and water excretion [30,31,32,33,34,35]; reduces renal oxidative stress by increasing PON2 expression and inhibiting NADPH oxidase activity [36]; interacts with other systems and receptors in the kidney [37,38]	Impaired D_1_-like receptor-mediated natriuretic and diuretic effects in hypertensive animal models [39,40,41]; impaired D_1_-like receptor-mediated inhibition of renal proximal sodium transport in human hypertensive subjects [42]; increased D_1_R serine phosphorylation and subsequent uncoupling of the D_1_R from its G protein/effector complex [43,44]; decreased renal D_1_R expression and aberrant interaction between D_1_R and other receptors in hypertension [37,38,45,46,47]
D_2_R (*DRD2*)	Renal proximal tubule, distal convoluted tubule, cortical and medullary collecting ducts	Synergistically interacts with D_1_R to inhibit renal sodium transport [48] and increases sodium excretion [49]; decreases renal ROS production [50]; increases the synthesis of dopamine in the kidney [51]	Decreased plasma membrane D_2_R expression in urine-derived RPT cells isolated from subjects with inverse salt sensitivity [52]; impaired renal D_1_-like and D_2_-like receptor interaction in SHRs [53]
D_3_R (*DRD3*)	Renal proximal tubule, thick ascending limb of Henle, distal convoluted tubule, cortical and medullary collecting ducts	Interacts with other GPCRs, e.g., AT_1_R [54], and D_5_R [55] and inhibits the expression and/or activities of renal sodium exchangers [56,57]; increases sodium excretion in WKY rats [58], salt-resistant Dahl rats fed normal or high-sodium diet and salt-sensitive Dahl rats fed normal-sodium diet [59]; regulates renal hemodynamics [60]	Decreased D_3_R expression in the renal cortex [61] and RPT cells [54] of SHRs, relative to WKY rats; impaired D_3_R agonist-induced increased D_3_R expression in RPT cells of SHRs [54]; impaired D_3_R-mediated natriuresis in SHRs [58]; aberrant interaction between D_3_R and other receptors in SHRs [54,55]
D_4_R (*DRD4*)	Renal proximal tubule, distal convoluted tubule, cortical and medullary collecting ducts	Increases sodium excretion and urine volume in Ang II-pretreated WKY rats [62]; inhibits vasopressin-dependent sodium transport and water permeability in the cortical collecting duct [63]; inhibits NKA activity in RPT cells [62]; interacts with other GPCRs, e.g., AT_1_R [62,64], insulin receptor [65] in the kidney	Increased D_4_R expression in the renal cortex of SHRs [61]; increased phosphorylation of D_4_R in SHR RPT cells [62]; impaired D_4_R-induced diuretic and natriuretic effects in SHRs [64]; aberrant interaction between D_4_R and other receptors in SHRs [62,64,65]
D_5_R (*DRD5*)	Renal proximal tubule, thick ascending limb of Henle, distal convoluted tubule, and cortical collecting duct	Inhibits renal sodium transport [32,55] and increases sodium and water excretion [66,67]; decreases renal oxidative stress by inhibiting NADPH oxidase [68]; interacts with other systems and receptors in the kidney [69]	Decreased D_5_R expression in SHR RPT cells and renal brush border membranes of SHRs [69]; male hD_5_R^F173L^ transgenic mice have increased blood pressure, decreased natriuresis and diuresis [67]; decreased Trx1 expression but increased NADPH oxidase activity, ROS generation, and AT_1_R expression in male hD5R^F173L^ mice [67]

**Table 2:** Effects of dopamine receptor modification on renal function and blood pressure.

Receptor Subtype	Receptor Modification	Animal Blood Pressure Phenotype	Receptor-Mediated Functions
D_1_R	Selective renal inhibition of D_1_R with AS-ODN	Systolic blood pressure is not affected by the renal infusion of AS-ODN *Drd1* in female SD rats fed normal or high salt diet [33]	Reduced urinary sodium and water excretion in AS-ODN *Drd1*-treated female SD rats fed normal or high salt diet [33]
	Global *Drd1* knockout mice	Increased systolic and diastolic blood pressures in *Drd1* knockout mice fed normal salt diet [34]	Impaired dopamine-mediated stimulation of cAMP production in homozygous *Drd1* knockout mice [34]
D_2_R	Homozygous global *Drd2* knockout mice	Increased systolic and diastolic blood pressures [79]; caused salt-dependent increase in blood pressure in male *Drd2* knockout mice [80]	Increased epinephrine excretion, sympathetic and ETB receptor activities, basal NKA activity in renal cortex and medulla, and urine flow and sodium excretion in *Drd2* knockout mice on normal-salt diet [79] but decreased sodium excretion on high-salt diet [80]
	Renal cortical *Drd2* depletion with siRNA	Increased systolic blood pressure [81]	Increased renal inflammation and injury [81]
D_3_R	Global *Drd3* knockout mice	Renin-dependent hypertension [87]; increased systolic and diastolic blood pressure on a normal salt diet [56]	Increased renal renin levels [87] and renal AT_1_R [54] and NHE3 [56] expressions; decreased urinary sodium excretion [54,87]
	Global *Drd3* knockout mice	Blood pressure not increased in male *Drd3* knockout mice, regardless of salt intake [88]	Decreased urinary sodium excretion on high-salt diet [88]
D_4_R	Global *Drd4* knockout mice	Increased systolic, diastolic, and mean blood pressures in both male and female *Drd4* knockout mice [95]; increased MAP on low, normal, and high salt diet, decreased sodium excretion and right-shifted pressure-natriuresis curve in male *Drd4* knockout mice [96]	Increased AT_1_R expression in renal homogenates and membranes [95]; increased expressions of NHE3, NKCC2, and NCC in the kidney, and increased expression of α-ENaC in the renal outer medulla on normal-salt diet; decreased expressions of renal NKCC2, NCC, α-ENaC, and α-NKA on low-salt diet; increased α-ENaC on high salt diet; increased NKCC2, NCC, α-ENaC, and α-NKA in renal plasma membrane on high salt diet [96]
	Renal cortical *Drd4* depletion with siRNA	Increased systolic blood pressure in male mice fed normal salt diet [97]	Increased renal NCC expression but unchanged urinary sodium excretion [97]
D_5_R	Global *Drd5* knockout mice	Increased systolic, diastolic, and mean blood pressures in mice fed normal sodium diet [68,69,102], and aggravated by a high sodium diet [68,102]	increased AT_1_R expression in the kidney of mice fed normal sodium diet [69,102]; increased expressions of NKCC2, NCC, and α and γ ENaC in the kidney of mice on normal and high sodium diet [102]; increased expressions of NHE3 and NaPi2 in the kidney of mice on high sodium diet [102]; increased renal NADPH oxidase protein expression and activity in the kidney of mice on normal sodium diet that is not affected by a high sodium diet [68]

## Data Availability

Data sharing is not applicable to this article because no data were generated or analyzed during this study.
